# Material-Dependent Toxic Mechanisms of Different Types of Particulate Emerging Contaminants Toward *Chlorella vulgaris*

**DOI:** 10.3390/toxics14060519

**Published:** 2026-06-15

**Authors:** Xiaona Li, Xiangjun Hou, Yu Kong, Ning Liu, Zhenyu Wang

**Affiliations:** 1 Institute of Environmental Processes and Pollution Control, School of Environment and Ecology, Jiangnan University, Wuxi 214122, China; xiaonali@jiangnan.edu.cn (X.L.);; 2 Stockbridge School of Agriculture, University of Massachusetts, Amherst, MA 01003, USA

**Keywords:** emerging contaminants, nanoplastics, metal/metal-oxides nanoparticles, toxic mechanisms, bioavailability

## Abstract

Particulate emerging contaminants (PECs) pose increasing ecological risks due to their widespread occurrence and complex environmental behaviors, yet their heterogeneous toxic mechanisms remain poorly understood, especially under environmentally relevant conditions and concentration gradients. Here, *Chlorella vulgaris* was used as a model organism to systematically compare the effects of polystyrene nanoparticles (PSNPs), silver nanoparticles (AgNPs), and titanium dioxide nanoparticles (TiO_2_NPs) across environmentally relevant and elevated concentrations (100 μg/L and 10 mg/L). Distinct toxicity pathways were identified among PEC types. PSNPs primarily induced chronic interference via particle–cell interactions, heteroaggregation, sedimentation-driven shading, and extracellular polymeric substance (EPS) regulation, rather than ROS-dominated toxicity. In contrast, AgNPs exhibited transformation-driven toxicity, undergoing intracellular speciation into Ag_2_S, AgCl, and Ag^+^, which triggered oxidative stress, membrane damage, and lipid peroxidation. TiO_2_NPs showed relatively high bioavailability and persistent oxidative stress effects. These results demonstrate that PEC toxicity evolves with particle type and concentration. Importantly, oxidative stress alone is insufficient to capture PEC ecotoxicity, which also involves the long-term impacts on algal behavior, sedimentation dynamics, and energy metabolism. This study provides mechanistic insights into PEC-induced algal toxicity and supports the source-oriented management of particulate pollutants in aquatic environments, particularly in hotspot scenarios such as wastewater discharge and sediment resuspension.

## 1. Introduction

With the continuous advance of industrialization and urbanization, the variety and quantity of pollutants released into the environment by human activities have increased substantially [1,2]. Among them, emerging contaminants are a broad class of substances that pose potential risks to environmental and human health but are not yet routinely included in monitoring or regulatory frameworks [3]. In recent years, increasing attention has been directed toward particulate emerging contaminants (PECs) in aquatic environments, including engineered nanomaterials, micro- and nanoplastics, and tire-wear particles [4]. Unlike conventional dissolved pollutants, PECs occur as solid particles, colloids, or particle aggregates, and their environmental behavior is controlled not only by chemical composition but also by particle size, morphology, density, surface charge, surface functional groups, and aggregation-settling properties [5]. As a result, their transport, transformation, fate, and bioavailability in aquatic environments are often more complex than those of dissolved contaminants. Owing to their small size, large specific surface area, and high interfacial reactivity, PECs can interact strongly with biological surfaces and may therefore pose considerable ecological risks [6]. PECs have been detected in rivers, lakes, groundwater, drinking-water sources, wastewater-treatment systems, and sediments, highlighting their widespread occurrence in aquatic ecosystems [7,8,9,10].

Importantly, PECs should not be treated as a single homogeneous class of pollutants. Different particulate materials can differ substantially in colloidal stability, ion-release potential, photochemical activity, and surface adsorption behavior, which in turn may lead to distinct environmental behavior and ecotoxicological effects [11]. For example, silver nanoparticles (AgNPs) are well known for their transformation potential and metal-ion release, titanium dioxide nanoparticles (TiO_2_NPs) for their relative stability and photocatalytic activity, and polystyrene nanoparticles (PSNPs) for their persistence and particle-specific interfacial effects [12,13,14]. These material-dependent properties can regulate particle dispersion, heteroaggregation with biota, sedimentation behavior, oxidative reactivity, and ultimately the bioavailability and toxicity in aquatic systems [15]. Therefore, understanding PEC toxicity requires not only evaluating whether these particles are harmful, but also clarifying how different particulate materials induce toxicity through different dominant pathways.

Microalgae are among the most widely used model organisms in aquatic ecotoxicology because they form the base of aquatic food webs, respond sensitively to environmental stress, and are relatively easy to cultivate under controlled conditions [16,17]. Previous studies have shown that PECs can affect algal growth and physiology through multiple pathways, including surface adsorption, heteroaggregation, shading effects, ion release, reactive oxygen species (ROS) generation, membrane damage, extracellular polymeric substance (EPS) responses, and metabolic disturbance [18,19,20,21]. However, despite substantial progress, current knowledge remains fragmented. Many studies have focused on single particle types, used different particle properties and exposure media, or applied concentrations far above realistic environmental levels, which reduces comparability across studies [22,23,24,25]. More importantly, systematic comparisons of common and distinct toxicity mechanisms among different PEC types under a unified experimental framework remain limited [26,27]. This study moves beyond conventional endpoint-based toxicity comparisons by systematically examining the mechanistic divergence among representative PEC classes under standardized exposure conditions. By coupling particle behavior with algal physiological responses, we aimed to identify not only whether different PECs induce algal toxicity, but also how their transformation potential, bioavailability, and interfacial interactions govern distinct toxicity pathways in microalgae. We therefore hypothesized that different PECs would exhibit material-dependent toxicity mechanisms toward microalgae, specifically that AgNPs, TiO_2_NPs, and PSNPs would differ in their effects on algal growth and physiology because of differences in ion release, surface reactivity, and particle–cell interfacial behavior, and that these differences would vary across exposure concentrations.

To test this hypothesis, we selected three representative PECs such as AgNPs, TiO_2_NPs, and PSNPs as model materials, representing metal nanoparticles, metal oxide nanoparticles, and plastic nanoparticles, respectively. *Chlorella vulgaris* (*C. vulgaris*), a typical freshwater primary producer and an ecologically relevant algal species in Taihu Lake, was used as the test organism. Taihu Lake, the third largest freshwater lake in China, is an important sink for multiple emerging contaminants, including nanoplastics and engineered nanomaterials, making it a relevant environmental context for exposure to PECs. Accordingly, we conducted exposure experiments at three concentration levels, including an environmentally relevant concentration based on Taihu Lake as well as 100 μg/L and 10 mg/L. We measured PEC bioavailability, algal growth rate, heteroaggregation, sedimentation behavior, photosynthetic pigment content, malondialdehyde (MDA), ROS production, cell-membrane damage, and EPS responses. By comparing the effects of different PEC types on algal growth and related physiological responses across concentration gradients, this study aimed to reveal material-dependent toxicity pathways and to provide a basis for the ecological risk assessment of representative PECs in freshwater environments.

## 2. Materials and Methods

### 2.1. Material Sources and Preparation

AgNPs (>99%) were purchased from Beijing Yijin New Material Technology Co., Ltd. (Beijing, China), and TiO_2_NPs (99.8%) were obtained from Shanghai Aladdin Biochemical Technology Co., Ltd. (Shanghai, China). Styrene (≥99%) was obtained from Beijing Innochem Science & Technology Co., Ltd. (Beijing, China). Sodium dodecyl sulfate (SDS, ≥99%), sodium chloride (NaCl, ≥99.9%), potassium ferricyanide (K_3_Fe(CN)_6_, ≥99%) and dichloromethane (DCM, ≥99.8%) were obtained from Shanghai Titan Scientific Co., Ltd. (Shanghai, China). Potassium persulfate (KPS, ≥99.5%) and sodium sulfide solution (Na_2_S, 99.9%) were obtained from Shanghai Macklin Biochemical Co., Ltd. (Shanghai, China). PSNPs were synthesized based on the reported methods [28]. Detailed information was shown in Text S1. *C. vulgaris* (strain FACHB-8) with the zeta potential of −14.37 ± 0.97 mV obtained from the Freshwater Algae Culture Collection (FACHB-Collection, Wuhan, China), Institute of Hydrobiology, Chinese Academy of Sciences. Nitric acid (HNO_3_, 65–68 wt%) were obtained from Sinopharm Chemical Reagent Co., Ltd. (Shanghai, China). Ultrapure water (resistivity 18.2 MΩ·cm^−1^) was prepared by Milli-Q Integral 3 (Merck Millipore, Darmstadt, Germany). Phosphate-buffered saline (PBS, 20X), sodium thiosulfate (Na_2_S_2_O_3_, 99%), ethylenediaminetetraacetic acid (EDTA, 99%), 2-morpholinoethanesulfonic acid (MES, ≥98%) and macerozyme R-10 (≥3000 U/g) were obtained from Shanghai Meryer Biochemical Technology Co., Ltd. (Shanghai, China).

### 2.2. Material Characterization

The crystal structures of AgNPs and TiO_2_NPs were analyzed by X-ray diffraction (XRD; D8 Advance, Bruker, Karlsruhe, Germany). The scanning range was 20–90° with a scan rate of 2°/min. Surface functional groups of PSNPs were characterized by fourier transform infrared spectroscopy (ATR-FTIR, Vertex 70, Bruker, Karlsruhe, Germany). A small amount of dried powder sample was placed on a clean and dry sample stage, compacted, and scanned in the range 4000–500 cm^−1^. The morphologies and particle sizes of AgNPs was characterized using a transmission electron microscope (TEM, JEM-2100F, JEOL, Tokyo, Japan). The morphological properties and particle sizes of TiO_2_NPs and PSNPs were determined using a scanning electron microscope (SEM, Regulus8100, Hitachi, Tokyo, Japan). The hydrodynamic diameter and surface charge of 10 mg/L AgNPs, TiO_2_NPs, and PSNPs in BG11 medium were determined using a Zetasizer (Nano-ZS90, Malvern Panalytical, Malvern, UK). The experiments were conducted at room temperature and the initial pH of the BG11 medium; the pH was not controlled after the addition of PECs (*n* = 3).

### 2.3. C. vulgaris Culture and PECs Exposure

*C. vulgaris* was cultured in BG11 medium. After preparation, the initial pH of the medium was 7.35. The medium was sterilized in an autoclave at 120 °C for 20 min, cooled to room temperature, irradiated with ultraviolet light for 30 min on a clean bench, and then inoculated with microalgae. PEC exposure was initiated after the microalgae reached the logarithmic growth phase. Cultures were maintained in an illuminated incubator at 4000 lux with a light:dark cycle of 12 h:12 h. The exposure experiments were conducted in 50 mL Erlenmeyer flasks containing 30 mL BG11 medium. A total of 30 flasks were used, including one control group (microalgae only, *n* = 3) and nine exposure groups covering three PEC types (AgNPs, TiO_2_NPs, and PSNPs) at three concentrations, with three biological replicates in each group. Each Erlenmeyer flask was shaken manually three times per day and randomly repositioned daily to ensure uniform illumination. After 10 days of exposure, growth-related indicators of algal cells were determined. The experiments were conducted under routine microalgal culture conditions (visible-light incubation and gentle manual shaking), which, according to previous studies, are not expected to cause the pronounced abiotic transformation of PECs compared with UV/simulated sunlight exposure or prolonged high-shear conditions. Therefore, abiotic controls were not included in the present study [29,30,31,32,33,34,35].

The exposure concentrations of AgNPs, TiO_2_NPs, and PSNPs were set to represent the environmental concentration in Taihu Lake, an intermediate effect concentration, and a toxic concentration. Specifically, the environmental concentrations were set at 100 ng/L for AgNPs, 10 μg/L for TiO_2_NPs, and 10 μg/L for PSNPs. The exposure levels were selected as environmental concentrations with reference to recently published data for Taihu Lake [36,37,38]. The toxic concentration was 10 mg/L and the intermediate concentration was 100 μg/L. Overall, the selected concentrations span low, near-threshold, and EC_50_-relevant/high-effect ranges depending on PEC type. Therefore, the present study was designed to compare the mechanistic differences among PEC classes under matched exposure conditions, rather than to determine material-specific toxicity thresholds [24,39,40,41,42,43,44,45,46,47].

### 2.4. Determination of Algal Toxicity Endpoints

#### 2.4.1. Growth Inhibition Rate

The optical density (OD_i_) of algal cells under different concentration gradients was measured at 680 nm using a UV spectrophotometer, and algal cell counts were determined by flow cytometry. The calibration equation was: cell density (1 × 10^6^ cells/mL) = 24.354 × OD_i_ − 0.0899 (R^2^ = 0.9997) (Figure S1). After exposure, OD values of each algal suspension were measured (with correction for the background absorbance of the corresponding PECs), and algal growth inhibition rates were calculated with the following Equation (1). Equation (1) was used to convert OD_i_ into cell density, and growth inhibition was subsequently calculated based on the relative difference in cell density between the treatment and control groups [48,49,50].

(1) $$Inhibitionratio\left(\%\right)=\frac{{OD}_{i}-{OD}_{CK}}{{OD}_{CK}}\times 100$$

where OD_i_ represents the optical density of algal cells measured at 680 nm under different PEC treatments, while OD_CK_ represents the optical density of the CK group at 680 nm.

#### 2.4.2. Heteroaggregation Behavior

At the initial stage of exposure, co-settling and total-settling curves for algal cells and PECs were measured. Settling curves over 120 min were first determined separately for algal cells alone and PECs alone; the sum of the OD values in algal cells alone and PECs alone treatments was the total-settling value, which was used to create the total-settling curve (indicated by ‘/’, e.g., “Algae/10 mg/L PSNPs”). The co-settling value of microalgae and PECs over 120 min was used to create the co-settling curve (indicated by ‘+’, e.g., “Algae + 10 mg/L PSNPs”). Settling curves were fitted with the following Equation (2):

(2) $$y=y_{0}+Pe^{-kt}$$

where *y*_0_ is the initial value of OD_t_/OD_0_ (OD_t_ is the absorbance of the algal suspension at time *t* and OD_0_ is the initial absorbance at *t* = 0), *P* is the decrease in OD_t_/OD_0_ when the curve reaches a stable state, *k* is the settling rate constant (OD/min), and *t* is settling time.

#### 2.4.3. Determination of PECs Bioavailability

The bioavailability of AgNPs inside algal cells was measured. Specifically, 15 mL of algal suspension was centrifuged at 4 °C and 8000 rpm for 10 min. The pellet was washed twice with a solution composed of 10 mM K_3_Fe(CN)_6_ and 10 mM Na_2_S_2_O_3_ (*v*:*v* = 1:1), then once with deionized water. The washed algal pellet was freeze-dried and weighed [51]. To determine total Ag, the freeze-dried pellet was transferred into a digestion tube, mixed with 3 mL HNO_3_ and 3 mL deionized water, digested by microwave at 190 °C and 1600 W. The detailed information in the determination of different Ag species according to previously reported methods [52] is shown in Text S2.

The bioavailability of TiO_2_NPs inside algal cells was determined. Specifically, 30 mL of algal suspension was centrifuged at 8000 rpm for 10 min. The pellet was washed sequentially with 0.1 M PBS and 0.02 M EDTA (twice and once, respectively), freeze-dried and weighed, then transferred into a digestion tube with 3 mL HNO_3_ and 3 mL deionized water and digested by microwave at 190 °C and 1600 W [53].

The bioavailability of PSNPs inside algal cells was measured. Specifically, 30 mL of algal suspension was centrifuged at 8000 rpm for 10 min, and the pellet was washed three times with 0.1 M PBS, freeze-dried, and weighed. Then 3 mL Tetramethylammonium hydroxide (TMAH) (25%, *v*/*v*) was added, and the sample was shaken for 24 h (25 °C, 150 rpm). After centrifugation and washing, 10 mL ethanol was added and the sample was kept in a water bath at 80 °C for 30 min. After full extraction, the mixture was centrifuged at 6000 rpm for 5 min, the precipitate was freeze-dried again, redissolved repeatedly in 5 mL DCM in a fume hood, concentrated, and carefully transferred into a 2 mL vial for storage [54].

#### 2.4.4. Algal-PECs Settling Experiment

After 10 days of PEC exposure, the suspended algal and PECs were sampled to conduct algal-PECs setting experiment. Detailed information is shown in Text S3.

#### 2.4.5. Determination of Algal Membrane Damage

Two milliliters of algal suspension was centrifuged at 4 °C and 4500 rpm for 5 min, the supernatant was removed, and the pellet was washed twice with 2 mL pre-cooled 0.1 M PBS under the same conditions. The pellet was then stained with 400 μL of 5 mg/L Propidium Iodide (PI, Nanjing Jiancheng, Nanjing, China), incubated at 25 °C in the dark for 30 min, washed once with 0.1 M PBS to remove surface dye, and the relative fluorescence intensity was measured at Ex/Em = 535/620 nm using a microplate reader.

#### 2.4.6. ROS Production

Two milliliters of algal suspension was centrifuged at 4 °C and 4500 rpm for 5 min, the supernatant was removed, and the pellet was washed twice with 2 mL pre-cooled 0.1 M PBS. The sample was then stained with 2 mL of 4 μM DCFH-DA (Nanjing Jiancheng, Nanjing, China), incubated at 37 °C in the dark for 30 min, washed once with 0.1 M PBS, and the relative fluorescence intensity was measured at Ex/Em = 488/525 nm.

#### 2.4.7. MDA Content and Superoxide Dismutase (SOD) Activity

Detailed information on the determination of MDA and SOD activity is shown in Text S4.

#### 2.4.8. EPS Analysis

Algal EPS constituents were explored using three-dimensional excitation-emission matrix spectroscopy (3D-EEM; F-7000, Hitachi, Tokyo, Japan) equipped with a 450 W xenon lamp at room temperature. Detailed information is shown in Text S5.

#### 2.4.9. Chlorophyll a and Carotenoids

Two milliliters of algal suspension was centrifuged at 4 °C and 4500 rpm for 5 min. After removal of the supernatant, 2 mL of 95% ethanol was added and pigments were extracted at 4 °C in the dark for 8 h. After centrifugation, the supernatant was transferred to a microplate and absorbance at 470 (OD_470_), 649 (OD_649_), and 665 (OD_665_) nm was measured to calculate chlorophyll a and carotenoid contents according to the following Equations (3) and (4):

(3) $$C_{chlorophyll\text{-}a}\left(mg/L\right)=13.7A_{665}-5.76A_{649}$$

(4) $$C_{carotenoids}\left(mg/L\right)=(1000A_{470}-2.05C_{chlorophyll\text{-}a})/245$$

where *C_chlorophyll-a_* represents the contents of chlorophyll-a, and *C_carotenoids_* represents the contents of carotenoids.

### 2.5. Data Processing and Analysis

All data are presented as mean ± standard deviation (Mean ± SD). Data were analyzed using Origin 2022b. Differences among treatments were tested by one-way analysis of variance (ANOVA) followed by the LSD test in SPSS 26 (*n* = 3, *p* < 0.05).

## 3. Results and Discussion

### 3.1. Morphological and Structural Characterization

TEM characterization revealed that AgNPs exhibited a spherical morphology (Figure S2a), with a primary particle size of 28.73 ± 8.25 nm. The corresponding hydrodynamic diameter was 413.44 ± 62.19 nm (Figure S3), and the zeta potential was −24.13 ± 1.46 mV (Figure S4).

SEM characterization showed that TiO_2_NPs possessed a typical rutile-phase structure (Figure S2b), with a primary particle size of 45.66 ± 10.23 nm. Their hydrodynamic diameter and zeta potential were determined to be 836.98 ± 60.50 nm (Figure S3) and −23.76 ± 1.56 mV (Figure S4), respectively. SEM images of PSNPs demonstrated uniformly distributed spherical particles (Figure S2c), with a primary particle size of 34.82 ± 6.35 nm. The hydrodynamic diameter was 623.53 ± 141.18 nm (Figure S3), and the zeta potential was −11.87 ± 1.41 mV (Figure S4).

The XRD pattern of AgNPs displayed characteristic diffraction peaks (Figure S5) at 2θ values of 38.1°, 44.3°, 64.5°, 77.4°, and 81.6°, which can be indexed to the (111), (200), (220), (311), and (222) crystal planes of face-centered cubic silver, respectively. For TiO_2_NPs, distinct diffraction peaks were observed at 2θ values of 27.5°, 36.0°, 41.3°, and 54.3°, corresponding to the (110), (101), (111), and (211) planes of rutile TiO_2_, confirming its crystalline phase. The ATR-FTIR spectrum of PSNPs exhibited characteristic absorption bands at 3027 cm^−1^, 2920/2850 cm^−1^, 1601/1493/1452 cm^−1^, 1028 cm^−1^, and 755/697 cm^−1^ (Figure S6), which are attributed to aromatic C–H stretching, aliphatic C–H stretching, aromatic C=C stretching, in-plane aromatic C–H bending, and out-of-plane aromatic C–H bending, respectively. Although PS-related bands and algal biochemical bands can both occur within the 400–1800 cm^−1^ fingerprint region, the spectra of exposed microalgal cells are generally similar to those of control cells, with the main differences reflected in subtle changes in peak intensity or band shape rather than in completely distinct spectral regions [39,55,56,57].

### 3.2. Toxicity Differences in PECs Toward C. vulgaris at Environmental Concentrations

**Figure 1:**
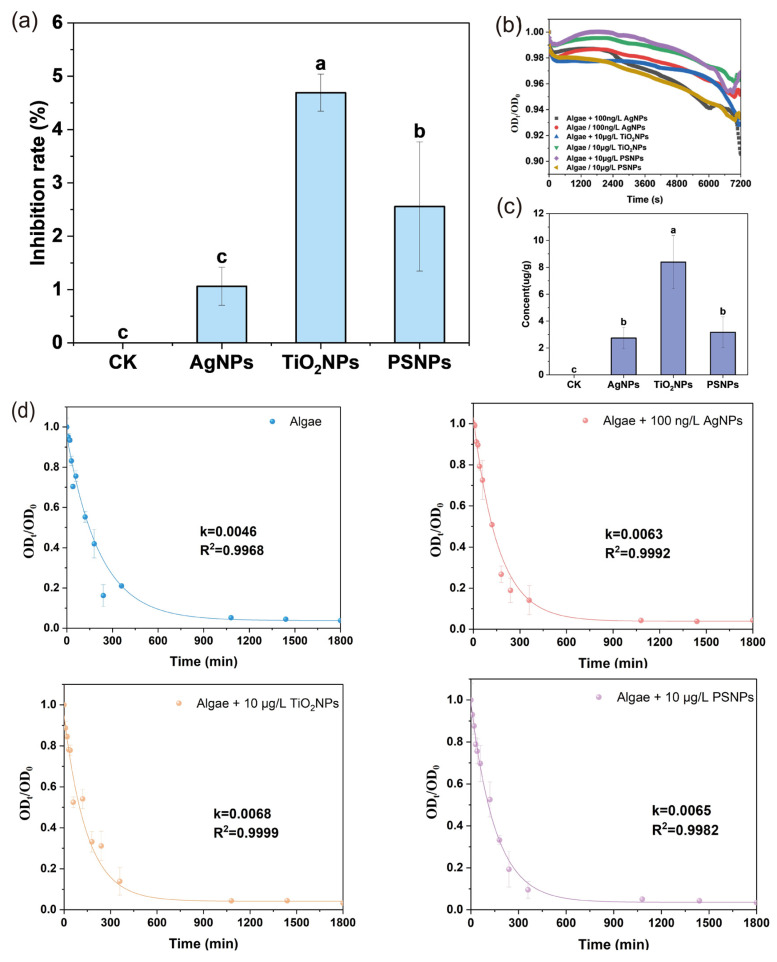
Environmental concentrations of PECs induced (**a**) algal growth inhibition, (**b**) heteroaggregation, (**c**) bioavailability, and (**d**) algal sedimentation. Different letter above each column represents significant differences among PECs treatments (*p* < 0.05).

At environmental concentrations, the inhibitory effects of the three PEC types on *C. vulgaris* were overall weak but already showed clear material-dependent differences. Compared with the control, growth inhibition caused by AgNPs, TiO_2_NPs, and PSNPs was 0.89%, 4.40%, and 1.13%, respectively (Figure 1a). The growth inhibition caused by PSNPs was intermediate between those of the two metal-based NPs. Combined with the heteroaggregation parameter, PSNPs showed the greatest heteroaggregation at the initial exposure stage (Figure 1b, Table S1), indicating that even at environmental concentrations PSNPs could establish a certain degree of surface contact with algal cells and co-settle with them. As PSNPs had the lowest absolute zeta potential, they could more readily overcome electrostatic repulsion from the algal-cell surface. Their stronger aggregation tendency likely enhanced physical contact with cells through settling or collision, which was also reflected by significantly increased bioavailability (Figure 1c), indicating more efficient physical contact and association with algal cells. Many studies have shown that nanoplastics can directly interfere with algal cells through physical processes such as adsorption and shading [19,39]. In addition to particle–cell heteroaggregation, previous studies suggest that stress-induced algal self-aggregation may also play a protective role by modulating external exposure and enhancing stress tolerance [58,59,60]. In this context, the enhanced surface contact and co-settling behavior of PSNPs observed here may not only increase particle association with algal cells, but also likely promote the self-aggregation of algal cells that partially buffered external stress [61,62]. Therefore, future research should distinguish between particle–cell heterogeneous aggregation and algal self-aggregation to better elucidate the mechanisms underlying the accelerated algal settling with PEC exposure.

In contrast, AgNPs did not significantly inhibit algal growth, indicating relatively weak toxicity. They had the lowest heteroaggregation parameter, suggesting that AgNPs were less likely to form complexes with algal cells at environmental concentrations, possibly because of their smaller hydrodynamic diameter and higher absolute zeta potential, which reduced adhesion to the cell surface [63]. TiO_2_NPs experienced stronger electrostatic repulsion yet the fastest settling rate (Figure 1d), consistent with their larger hydrodynamic diameter. This reduced suspension stability but may also have increased the probability of the particle coverage, local contact, and shading of algal cells; such contact may facilitate entry into cells by damaging the membrane, thereby increasing bioavailability.

**Figure 2:**
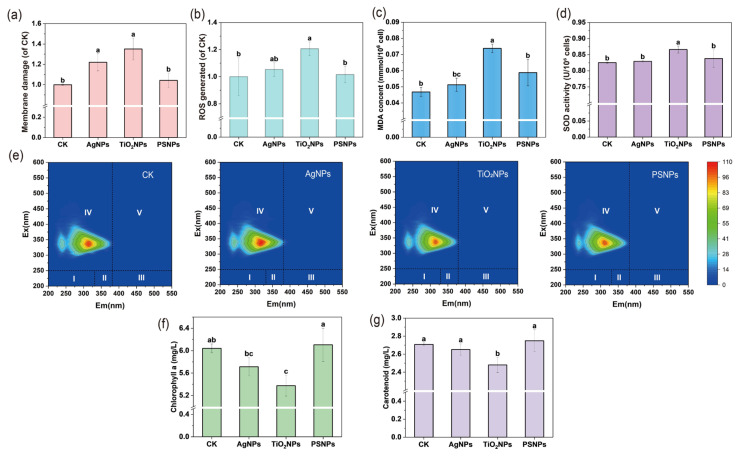
Responses of *C. vulgaris* induced by different PECs at environmental concentrations including (**a**) membrane damage, (**b**) ROS level, (**c**) MDA content, (**d**) SOD activity, (**e**) EPS constituents, (**f**) chlorophyll a content, and (**g**) carotenoids content. Different letter above each column represents significant differences among PECs treatments (*p* < 0.05).

At environmental concentrations, membrane damage caused by AgNPs, TiO_2_NPs, and PSNPs increased by 22.01%, 35.13%, and 4.28% relative to the control, respectively; ROS increased by 5.30%, 20.68%, and 1.56%; MDA content increased by 9.40%, 19.87%, and 2.78%; and SOD activity increased by 0.50%, 4.93%, and 1.54% (Figure 2a–d). Although oxidative-stress-related indicators in the PSNP treatment showed slight upward trends, none reached statistical significance (Figure 2a–d), indicating that PSNPs did not induce severe oxidative stress or membrane lipid peroxidation. Their toxicity therefore likely did not rely on a free-radical-damage mechanism, but rather on external-contact interference such as adhesion to the algal-cell surface and the alteration of the growth microenvironment [64].

**Figure 3:**
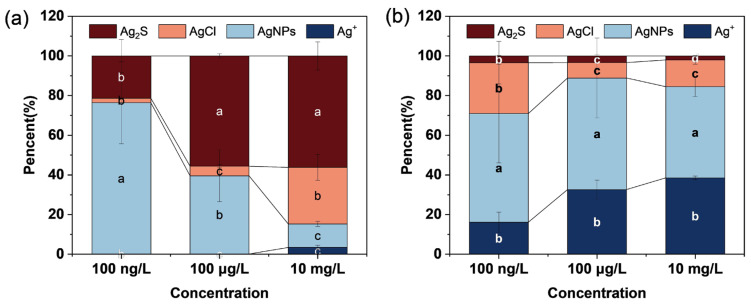
Transformation of AgNPs in algal cells. (**a**) Algal intracellular environment; (**b**) Algal extracellular environment. Different letter among each column represents significant differences in silver species among treatments (*p* < 0.05).

By contrast, although AgNPs did not show significant growth inhibition at environmental concentrations, they significantly induced ROS production in algal cells (an increase of 5.30%). At this stage, intracellular Ag was still present mainly as AgNPs (Figure 3a), indicating little transformation and suggesting that at this concentration AgNPs may primarily damage cells by altering membrane permeability.

The TiO_2_NP treatment triggered the most pronounced oxidative-stress response in *C. vulgaris*, including the strongest ROS production (20.68% increase), significant membrane damage (35.13% increase), and a synchronous increase in SOD activity (4.93% increase), indicating the activation of a typical antioxidant defense program. MDA content was also highest (19.87% increase), consistent with the strongest ROS signal, indicating severe lipid peroxidation [65].

Three-dimensional EEM was used to characterize EPS extracts from *C. vulgaris* under different treatments. The fluorescence results showed that the red-peak fluorescence intensity in the PSNP treatment was lower than that in the control (Figure 2e). Given the strong heteroaggregation of PSNPs, this suggests that, at environmental concentrations, PSNPs bound strongly with both cell surfaces and extracellular secretions, thereby changing the occurrence state and fluorescence characteristics of EPS [28]. AgNP exposure promoted the release or accumulation of fluorescent EPS components [66], whereas the decreased EPS peak under TiO_2_NP exposure may be attributable to its strong adsorption and settling behavior, implying that extracellular secretions were adsorbed by particles and then settled out.

Under PSNP exposure, photosynthetic pigment contents did not differ significantly from those in the control (Figure 2f,g), indicating that photosynthesis remained functional and was able to compensate for environmental disturbance caused by PSNPs. Thus, although PSNPs had already imposed some growth inhibition, they did not suppress chlorophyll a content. Previous studies have shown that under mild stress, microalgae may exhibit compensatory photosynthetic regulation to maintain basic physiological function [67]. In contrast, TiO_2_NPs caused the greatest decrease in both photosynthetic pigments (11.01% and 8.44%, respectively), which was highly consistent with their strong growth inhibition and elevated ROS and MDA, indicating a more direct impairment of the photosynthetic system [68]. AgNPs also reduced both pigments (by 5.43% and 2.13%, respectively), although less strongly than TiO_2_NPs, suggesting that pigment systems were already affected at low concentrations.

### 3.3. Toxicity Differences in PECs Toward C. vulgaris at 100 μg/L

**Figure 4:**
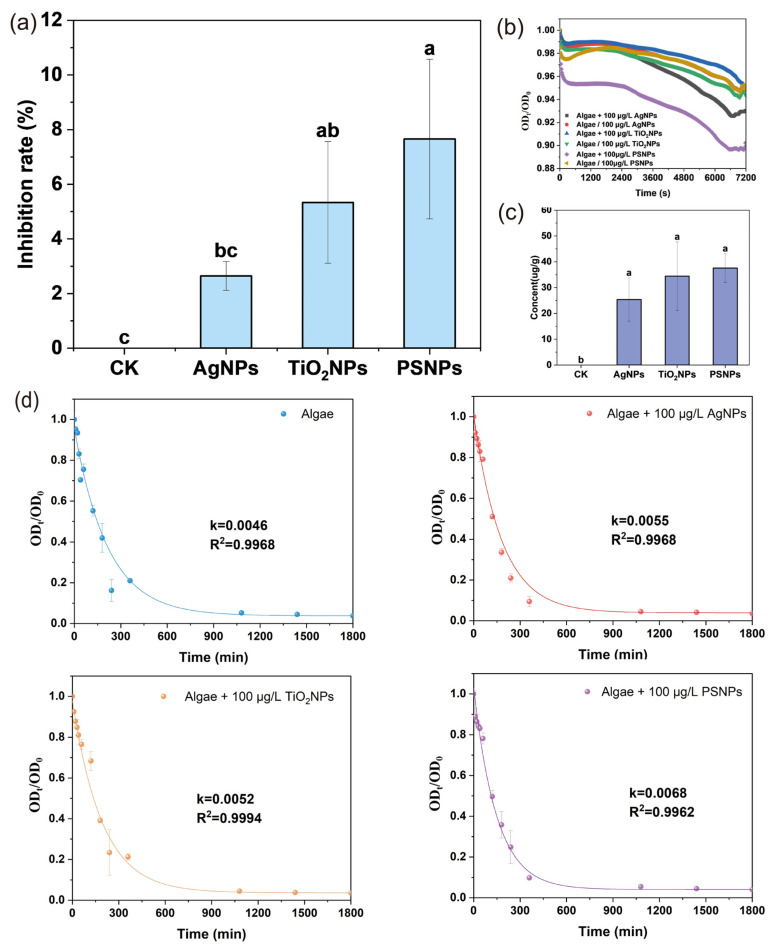
100 μg/L PECs induced the (**a**) algal growth inhibition, (**b**) heteroaggregation, (**c**) bioavailability, and (**d**) algal sedimentation. Different letter above each column represents significant differences among PECs treatments (*p* < 0.05).

When the concentration increased to 100 μg/L, the toxicity differences among PEC types were markedly amplified and the order of inhibition changed. Growth inhibition by the three PECs was 2.47%, 5.04%, and 7.44%, respectively (Figure 4a). In particular, growth inhibition by PSNPs increased significantly and became significantly higher than that of the control, indicating the substantial suppression of algal growth at this concentration. At the same time, the heteroaggregation of PSNPs was not pronounced (Figure 4b). Combined with their relatively low absolute zeta potential, which lies within an unstable range [39], this suggests that PSNP self-aggregation may have increased at this concentration, weakening heteroaggregation with algal cells. Nevertheless, PSNPs showed a markedly faster settling rate than AgNPs and TiO_2_NPs and maintained relatively high bioavailability (Figure 4b), perhaps because under prolonged exposure in algal suspensions they still contacted algal cells over time and formed complexes through long-term dispersed contact rather than through instantaneous settling-dominated interactions, and may have subsequently enter cells via passive uptake [69].

For AgNPs, increased concentration likely promoted further ion release, making them more prone to aggregate with negatively charged algal cells. Their heteroaggregation rate constant increased sharply to the highest level, which may be related to the instability of surface coatings or charge neutralization caused by ion release. The strong increase in aggregation likely enhanced efficient contact with algal cells and thus increased bioavailability [70]. Intracellular silver was dominated by Ag_2_S (Figure 3), indicating Ag^+^ release followed by sulfidation, which suggests that AgNPs indeed exerted toxic effects on algal cells and that the cells were attempting detoxification [71]. TiO_2_NPs remained highly toxic, but their settling rate became slower, possibly because aggregate structure and suspension state changed at this concentration [72].

**Figure 5:**
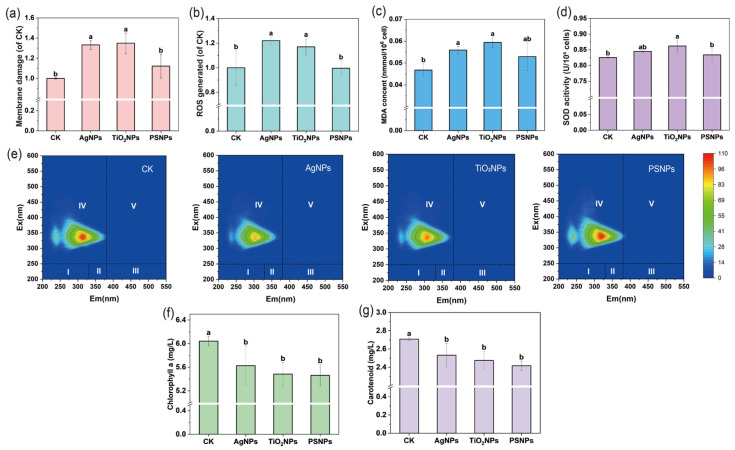
Responses of *C. vulgaris* of different PECs at 100 μg/L including (**a**) membrane damage, (**b**) ROS level, (**c**) MDA content, (**d**) SOD activity, (**e**) EPS constituents, (**f**) chlorophyll a content, and (**g**) carotenoids content. Different letter above each column represents significant differences among PECs treatments (*p* < 0.05).

At 100 μg/L, membrane damage caused by AgNPs, TiO_2_NPs, and PSNPs increased by 33.19%, 34.98%, and 12.18%, respectively relative to the control; ROS increased by 22.18%, 16.95%, and 13.99%; MDA content increased by 19.23%, 21.58%, and 6.62%; and SOD activity increased by 2.40%, 4.45%, and 1.04% (Figure 5a–d). The PSNP treatment still did not induce significant membrane damage, ROS generation, MDA accumulation, or SOD response, indicating that even at this concentration it did not trigger a classical oxidative-stress mechanism. This further supports the view that growth inhibition by PSNPs was not driven by disturbance of the oxidative-stress system, in sharp contrast to AgNP and TiO_2_NP treatments, for which oxidative stress was central. After entering algal cells, AgNPs may have undergone substantial sulfidation to form Ag_2_S, but at higher concentrations the coexistence of Ag^+^, AgCl, and other transformation products may have continued to disturb the intracellular redox balance (Figure 3), resulting in significantly elevated ROS [73]. The TiO_2_NP treatment continued to induce severe oxidative stress and strong membrane damage.

Three-dimensional fluorescence analysis further showed that different PEC treatments significantly altered the fluorescence-response characteristics of extracellular EPS (Figure 5e). Under PSNP stress, algal cells still maintained strong metabolic activity and released EPS to alleviate particle-attachment stress, thereby promoting the formation of stable PSNP–microalgae complexes [74]. The red-peak fluorescence intensity in the AgNP treatment was the lowest, indicating that under aggravated oxidative stress and membrane damage, extracellular secretion capacity declined, or that released organic matter had already participated in complexation reactions with AgNPs and their transformation products, thus changing fluorescence responses [75]. The TiO_2_NP treatment also showed lower fluorescence than the control, which may be related to the adsorption of extracellular organic matter and heteroaggregation. EPS can promote surface heteroaggregation between TiO_2_NPs and algal cells; once the complexes settle, fluorescence intensity in the aqueous phase decreases [76,77].

AgNP, TiO_2_NP, and PSNP treatments reduced chlorophyll a by 6.84%, 9.22%, and 9.57%, respectively, and reduced carotenoids by 6.53%, 6.83%, and 9.42%, respectively (Figure 5f,g). For PSNPs, the possible photosynthetic compensation observed at environmental concentrations disappeared and shifted to the significant inhibition of photosynthetic pigments, consistent with the marked increase in growth inhibition. This indicates that when contact pressure and particle burden increased, PSNPs began to affect the stability of the photosynthetic system. AgNP and TiO_2_NP treatments also significantly reduced photosynthetic pigments, consistent with the increased oxidative stress they imposed on algal cells [78].

### 3.4. Toxicity Differences in PECs Toward C. vulgaris at 10 mg/L

**Figure 6:**
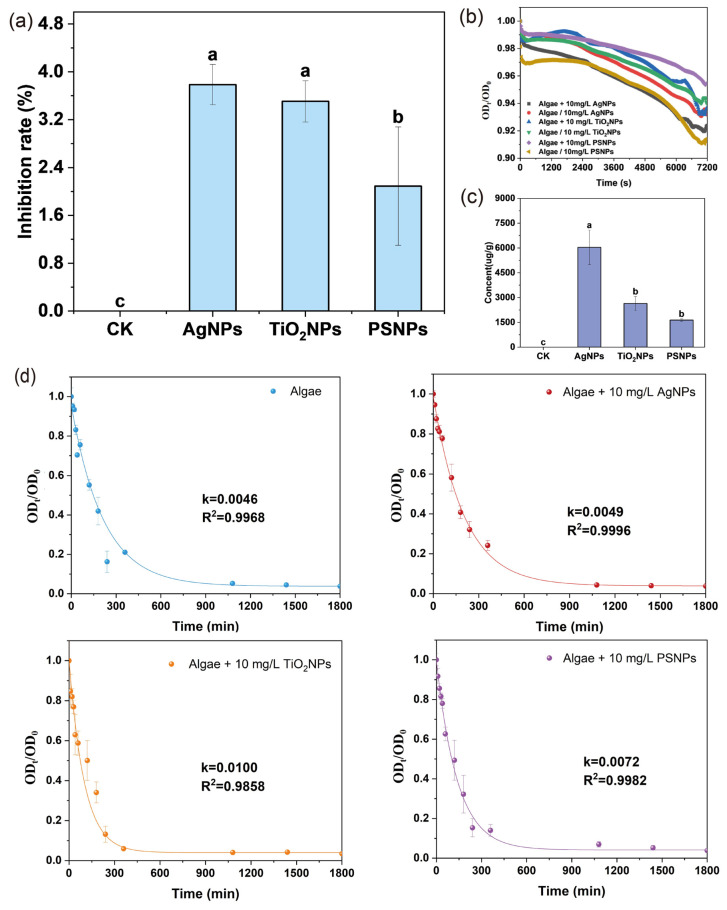
10 mg/L PECs induced the (**a**) algal growth inhibition, (**b**) heteroaggregation, (**c**) bioavailability, and (**d**) algal sedimentation. Different letter above each column represents significant differences among PECs treatments (*p* < 0.05).

Under high-concentration exposure at 10 mg/L, all three PECs significantly inhibited *C. vulgaris* growth, with inhibition rates of 3.56%, 3.21%, and 1.88%, respectively (Figure 6a). The order of toxicity changed again, and PSNPs showed the weakest inhibitory effect among the three, although still significantly higher than the control, indicating that even under extremely high exposure, the toxicity intensity of PSNPs remained relatively limited. At this concentration, the heteroaggregation parameter of the PSNP treatment remained the lowest (Figure 6b), likely because their low absolute zeta potential favored self-aggregation at high concentration. However, PSNP bioavailability remained significantly higher than in the control (Figure 6c), indicating that under prolonged high-concentration exposure, PSNPs may enter algal cells through internalization and sedimentation-related interference. Their high settling rate suggests that their effects may occur more through the disturbance of the algal-suspension environment [79].

The toxicity of AgNPs toward *C. vulgaris* was significantly enhanced at 10 mg/L, and their bioavailability was the highest. Their increased heteroaggregation indicates that they more readily formed complexes with algal cells, thereby aggravating structural damage and toxic responses. In addition, AgNPs continued to transform and release Ag^+^ at a high concentration, and intracellular Ag occurred mainly as Ag_2_S, indicating pronounced biotransformation (Figure 3). Membrane damage caused by Ag^+^ and the larger distribution of Ag^+^ in the extracellular medium suggest that, at high concentration, AgNP toxicity shifted toward an Ag^+^-dominated mechanism [80,81]. TiO_2_NPs also remained significantly toxic at high concentration. Their heteroaggregation increased, making them more likely to form complexes with algal cells and thereby more strongly affect particle aggregation, suspension, and sedimentation behavior, leading to enhanced membrane damage and toxicity [82].

**Figure 7:**
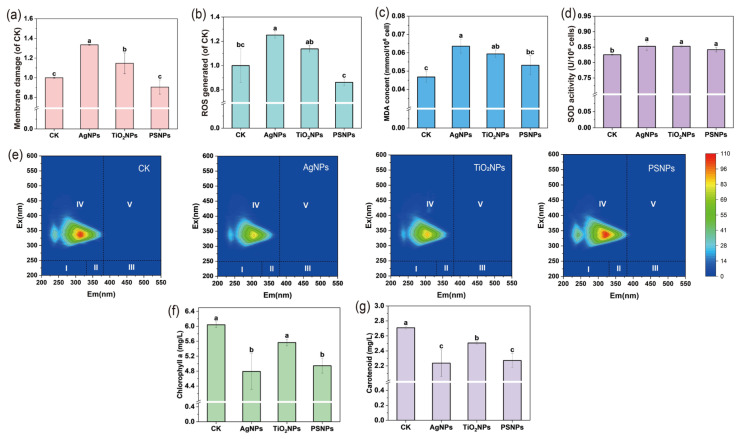
Responses of *C. vulgaris* of different PECs at 10 mg/L including (**a**) membrane damage, (**b**) ROS level, (**c**) MDA content, (**d**) SOD activity, (**e**) EPS constituents, (**f**) chlorophyll a content, and (**g**) carotenoids content. Different letter above each column represents significant differences among PECs treatments (*p* < 0.05).

The PSNP treatment still did not significantly alter membrane permeability or induce marked oxidative damage at this concentration (Figure 7a–d), further reinforcing that PSNP toxicity did not depend on oxidative stress. However, increased SOD activity indicated that PSNPs still imposed stress and activated defense mechanisms, without causing irreversible damage. Previous studies have shown that the effects of PSNPs on microalgae are strongly dependent on the concentration and exposure scenario; under some conditions, they mainly manifest as surface coverage, particle contact, and environmental interference without necessarily causing significant membrane damage or lipid peroxidation [83].

AgNPs caused the most severe membrane damage and strongly induced oxidative-stress responses. Relative to the control, ROS, MDA, and SOD increased by 25.19%, 48.50%, and 3.31%, respectively, indicating that toxicity was substantially amplified and had developed into irreversible oxidative damage. At high concentration, AgNP toxicity is often closely related to particle–cell contact, internalization, and Ag^+^ release. Intracellular silver mainly occurred as Ag_2_S and AgCl (Figure 3), indicating continuous sulfidation and chlorination after entry into the cellular system. Ag^+^ toxicity became more prominent at high concentration, and membrane disruption allowed more Ag^+^ to dissociate into the algal medium, so the coexistence of multiple products disturbed cellular homeostasis [84].

For TiO_2_NPs, membrane damage, ROS level, MDA content, and SOD activity increased by 14.70%, 13.74%, 17.09%, and 3.33%, respectively, indicating the maintenance of a significant ‘oxidative stress-membrane damage’ toxic mode. Many studies have shown that TiO_2_NPs can induce massive ROS generation, damage cell membranes, and alter antioxidant-enzyme activity, thereby causing lipid peroxidation and persistent physiological injury [85,86].

EPS fluorescence intensity was highest under PSNP exposure, indicating that even under high PSNP concentration *C. vulgaris* maintained a strong EPS secretion response (Figure 7e). EPS may form a defensive layer or local buffering matrix on the cell surface to mitigate the particle stress caused by PSNPs. This is consistent with the lack of significant membrane damage and ROS induction, suggesting that PSNPs toxicity mainly prompts extracellular defense and interfacial adaptation rather than ROS-dominated injury [74]. EPS fluorescence was lowest in the AgNP treatment, indicating that under strong oxidative damage and membrane disruption, extracellular secretion capacity may have been suppressed, or secreted substances had already participated extensively in complexation/adsorption processes, thereby weakening fluorescence signals [87]. The TiO_2_NP treatment was lower than the control for reasons similar to those at 100 μg/L.

AgNP, TiO_2_NP, and PSNP treatments reduced chlorophyll a by 20.68%, 7.88%, and 18.13%, respectively, and reduced carotenoids by 17.43%, 7.53%, and 16.13%, respectively (Figure 7f,g). The significant decline in photosynthetic pigments under the PSNP treatment indicates that at high concentrations, long-term particle contact, surface coverage, and settling clearly affect photosynthetic-system stability. AgNPs caused the largest decline in pigments, suggesting the strongest interference with the photosynthetic pigment system under high concentration, likely through the combined effects of particle–cell contact, Ag^+^ release, and oxidative stress, consistent with high ROS, high MDA, and strong membrane damage. TiO_2_NPs also decreased photosynthetic pigments, but overall inhibition was weaker than that of AgNPs and PSNPs. Previous studies have reported that the degree of inhibition of chlorophyll and carotenoids by TiO_2_NPs varies with concentration, light conditions, and particle aggregation state [88].

## 4. Conclusions

The three classes of PECs exhibited clearly different toxic mechanisms toward *C. vulgaris*. Overall, PSNP toxicity was not mainly expressed as typical oxidative injury, but rather as chronic interference driven by particle–cell interfacial interactions. Across different concentrations, PSNPs showed relatively strong heteroaggregation, settling, and bioavailability, accompanied by enhanced EPS release and reduced photosynthetic pigments, indicating that PSNPs more likely inhibited algal growth and photosynthetic function through surface attachment, particle coverage, local mass-transfer limitation, and extracellular microenvironment reconstruction. Especially at medium and high concentrations, the inhibitory effect of PSNPs increased, whereas ROS, membrane damage, and MDA did not increase synchronously, indicating a toxic mode dominated by physical contact and metabolic disturbance rather than by typical ROS-driven toxicity.

In contrast, AgNP toxicity showed a typical transformation-driven pattern. During exposure, AgNPs underwent marked speciation transformation mainly into Ag_2_S, AgCl, and Ag^+^, while continuously increasing ROS, membrane damage, MDA accumulation, and SOD responses, indicating that toxicity was primarily derived from particle internalization, transformation processes, enhanced oxidative stress, and aggravated membrane lipid peroxidation. TiO_2_NPs, as relatively stable metal-oxide NPs, exhibited large hydrodynamic diameters and fast settling rates, and their toxicity combined physical settling effects with oxidative injury. On the one hand, TiO_2_NPs readily formed large aggregates, contacted algal cells, settled with them, and entered cells; on the other hand, they continuously induced high ROS, membrane damage, MDA, and SOD responses while suppressing photosynthetic pigments, indicating a toxic mechanism characterized mainly by relatively high bioavailability and sustained oxidative stress.

To further clarify PECs toxic mechanisms, future studies should pay more attention to co-exposure with multiple pollutants, long-term low-dose exposure, the self-aggregation of algal cells, and PEC transformation and combined toxicity under realistic environmental media. Additional analyses of algal metabolites, other antioxidant-system endpoints, and microscopic imaging would also help improve the robustness of experimental results and further reveal the intrinsic links among particle–cell interfacial interactions, oxidative damage, and photosynthetic inhibition. This study highlights that PEC toxicity cannot be generalized and should be evaluated within a material-specific mechanistic framework.

## Data Availability

The data presented in this study are available on request from the corresponding author.

## Supplementary Materials

The following supporting information can be downloaded at: https://www.mdpi.com/article/10.3390/toxics14060519/s1, Text S1. Synthesis of PSNPs; Text S2. Determination of different Ag species; Text S3. Algal settling experiment; Text S4. Determination of MDA and SOD activity; Text S5. Analysis of EPS constituents; Figure S1. Standard curve of cell density optical density of *C. vulgaris*; Figure S2. Morphology and particle-size distribution of three PECs. (a) TEM image and particle-size distribution of AgNPs; (b) SEM image and particle-size distribution of TiO_2_NPs; (c) SEM image and particle-size distribution of PSNPs; Figure S3. Hydrodynamic diameter of PECs; Figure S4. Zeta potential of PECs; Figure S5. XRD patterns of AgNPs and TiO_2_NPs; Figure S6. ATR-FTIR spectrum of PSNPs; Table S1. Heterogeneous aggregation parameters of different PECs.
