# Supplementary material for: Material-Dependent Toxic Mechanisms of Different Types of Particulate Emerging Contaminants Toward Chlorella vulgaris
Source: Toxics. 2026 Jun 15;14(6):519. doi: 10.3390/toxics14060519 (PMC13308118; doi:10.3390/toxics14060519)
Supplement: Supplementary file 1 [file toxics-14-00519-s001.zip › toxics-4256500-supplementary.pdf]

Supplementary Material for

Material-Dependent Toxic Mechanisms of Different Types of Particulate Emerging Contaminants toward *Chlorella vulgaris*

Xiaona Li [1,2], Xiangjun Hou [1], Yu Kong [1,2], Ning Liu [1], Zhenyu Wang [1*]

1. Institute of Environmental Processes and Pollution Control, School of Environment and Ecology, Jiangnan University, Wuxi 214122, China

2. Stockbridge School of Agriculture, University of Massachusetts, Amherst, Massachusetts 01003, United States

*Correspondence to: Zhenyu Wang

E-mail address: wang0628@jiangnan.edu.cn

Content: 5 Texts, 6 Figures, 1 Table, 15 Pages

Text S1. Synthesis of PSNPs

Text S2. Determination of different Ag species

Text S3. Algal settling experiment

Text S4. Determination of MDA and SOD activity

Text S5. Analysis of EPS constituents

Figure S1. Standard curve of cell density optical density of *C. vulgaris*

Figure S2. Morphology and particle-size distribution of three PECs. (a) TEM image and particle-size distribution of AgNPs; (b) SEM image and particle-size distribution of TiO_2NPs; (c) SEM image and particle-size distribution of PSNPs.

Figure S3. Hydrodynamic diameter of PECs

Figure S4. Zeta potential of PECs

Figure S5. XRD patterns of AgNPs and TiO_2NPs

Figure S6. ATR-FTIR spectrum of PSNPs

Table S1. Heterogeneous aggregation parameters of different PECs

Reference

Text S1 Synthesis of PSNPs

Styrene was first purified by vacuum distillation. A mixture containing 7.8% styrene, 2% SDS, and 90.2% of 3.6 mM KPS was dissolved in deionized water and heated for 24 h at 70°C under nitrogen protection with stirring at 425 rpm. The resulting white emulsion was transferred into a dialysis bag (1 kDa) to remove residual SDS and other impurities generated during PSNPs synthesis. The purified emulsion was then freeze-dried under vacuum to obtain solid PSNPs. A dispersed PSNPs suspension was prepared by treatment in a water-bath ultrasonic processor at 100 kW/h for 3 h [1].

Text S2 Determination of different Ag species

To determine different Ag species, the algal pellet obtained by centrifugation was first weighed, then mixed with 4.8 mL MES buffer (25 mM, pH = 5.0). After 5 min of sonication, 8 mL MES buffer and 16 mL macerozyme R-10 (5%) were added. The mixture was shaken at 37°C for 24 h to fully disrupt the cell wall, and then centrifuged at 8,000 rpm for 10 min. The supernatant was collected in batches for speciation analysis [2]. To determine the dissolved Ag^+, 8 mL of supernatant was centrifuged at 4°C and 8000 rpm and filtered through a 0.22 μm aqueous membrane; to determine the dissolved Ag^+, AgNPs, and AgClNPs, 8 mL of supernatant was directly filtered through a 0.22 μm aqueous membrane; to determine the dissolved Ag^+ and AgClNPs, 8 mL of supernatant was mixed with 200 μL of ammonia, sonicated for 30 min, vortexed, centrifuged again, and the supernatant was filtered through a 0.22 μm aqueous membrane and stored in a 10 mL tube; to determine Ag_2SNPs, the pellet remaining after centrifugation was washed three times with Na_2S solution (pH = 7; 4, 4, and 2 mL, respectively). Before each wash, the sample was vortexed, sonicated, and centrifuged. The three wash solutions were combined, filtered through a 0.22 μm aqueous membrane, and stored in a 10 mL tube. To determine different extracellular Ag species in AgNPs-treated algal suspensions, the washed solution was centrifuged at 4°C and 8,000 rpm, and the supernatant was collected for the determination of dissolved Ag^+, dissolved Ag^+ + AgNPs + AgClNPs, dissolved Ag^+ + AgClNPs, and Ag_2SNPs using the same procedures described above.

Text S3 Algal settling experiment

At 0, 10, 20, 30, 40, 60, 120, 180, 240, 360, 720, 1440, and 1800 min, the upper-layer algal suspension was collected into a microplate and the absorbance at 680 nm was measured in real time. PECs settling curves were fitted with the following equation S1:

$$y = y_0 + Pe^{-kt} \quad \text{(equation S1)}$$

where y_0 is the initial value of A_t/A_0, P is the decrease in A_t/A_0 when the curve stabilizes, k is the settling-rate constant, and t is settling time.

Text S4 Determination of MDA and SOD activity

MDA reaction solution was prepared from 5.00% trichloroacetic acid containing 0.50% thiobarbituric acid (TBA). A total of 250 μL MDA extract and 250 μL MDA reaction solution were mixed in a 2 mL tube, vortexed thoroughly, heated in boiling water at 95°C for 40 min, cooled, and centrifuged at 4,500 rpm for 10 min. The supernatant was transferred to a microplate, and absorbance at 532 and 600 nm was measured using 0.1 M PBS as the blank.

SOD activity was also measured as follows: 250 μL reaction solution and 250 μL extract were mixed in a 2 mL tube and incubated in a light incubator for 20 min. Meanwhile, light-treated and dark-treated controls were prepared (control 1: 250 μL reaction solution plus the same volume of PBS, exposed to light for 20 min; control 2: PBS incubated in the dark for 20 min). After color development, absorbance at 560 nm was measured in the dark using a microplate reader, with control 2 used for zeroing.

Text S5 Analysis of EPS constituents

Algal suspension was centrifuged at 4°C and 4,500 rpm for 10 min, then 1.5 mL of 6% NaCl solution was added and centrifuged at 4°C and 10,000 rpm for 30 min. The supernatant was collected and filtered. Three-dimensional excitation-emission matrix spectroscopy (3D-EEM; F-7000, Hitachi, Japan) equipped with a 450 W xenon lamp was used at room temperature. Excitation wavelengths ranged from 200 to 550 nm (5 nm interval) and emission wavelengths from 200 to 600 nm (5 nm interval), with a scan speed of 12,000 nm/min and detector voltage of 400 V.

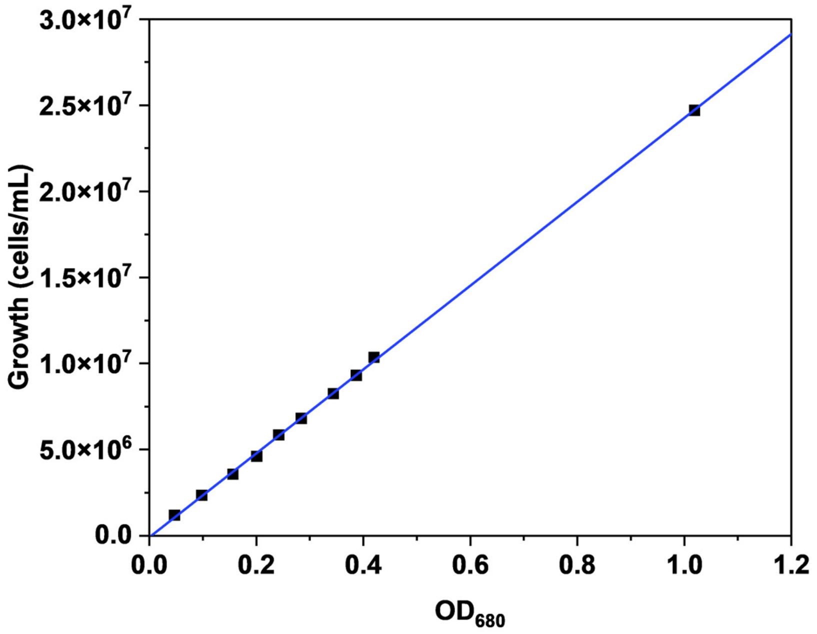

Figure S1 Standard curve of cell density optical density of *C. vulgaris*

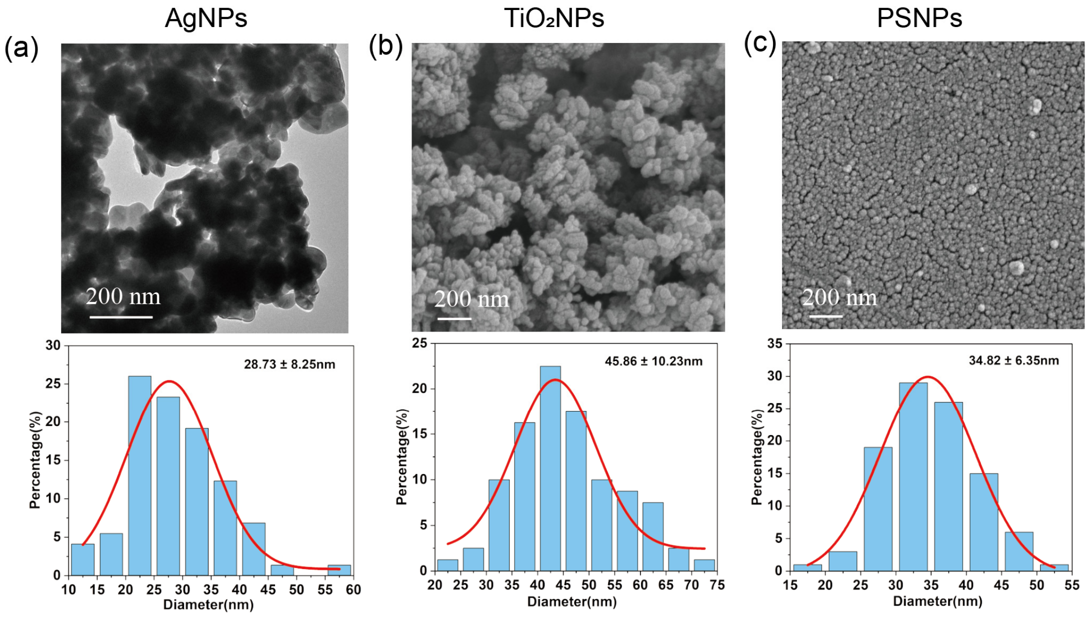

Figure S2. Morphology and particle-size distribution of three PECs. (a) TEM image and particle-size distribution of AgNPs; (b) SEM image and particle-size distribution of TiO_2NPs; (c) SEM image and particle-size distribution of PSNPs.

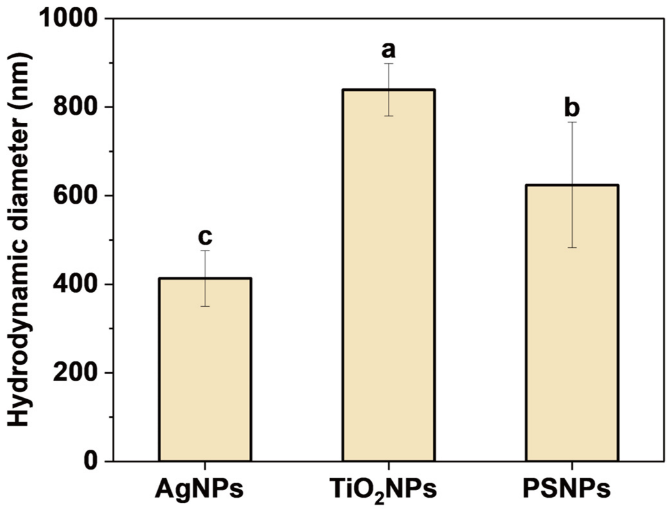

Figure S3. Hydrodynamic diameter of PECs

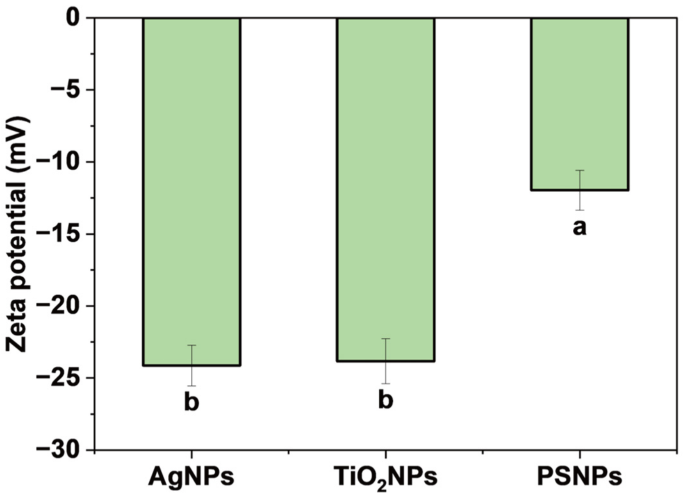

Figure S4. Zeta potential of PECs

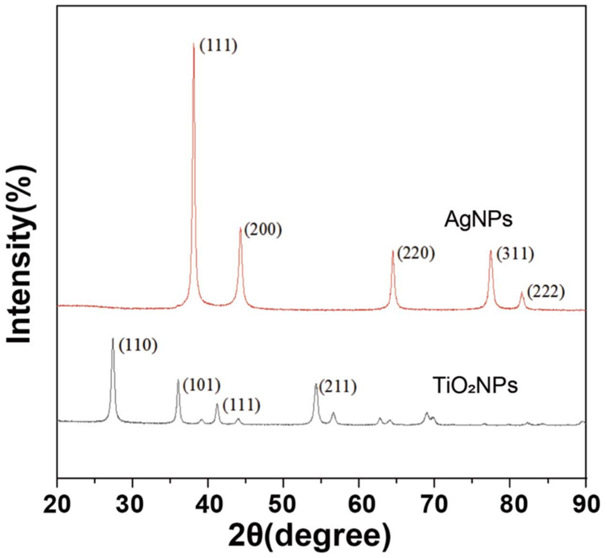

Figure S5. XRD patterns of AgNPs and TiO_2NPs

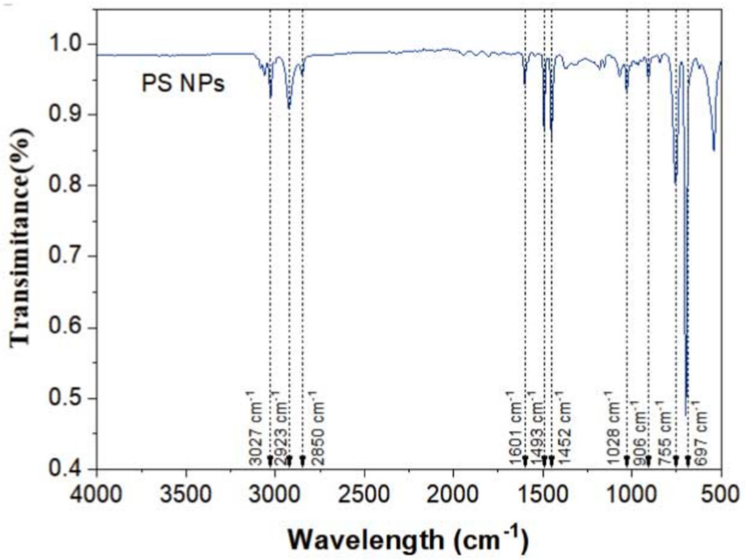

Figure S6. ATR-FTIR spectrum of PSNPs

Table S1 Heterogeneous aggregation parameters of different PECs

**Concentrations**	**AgNPs**	**TiO_2NPs**	**PSNPs**
100ng/L-10μg/L	0.00984	0.00133	0.01408
100 μg/L	0.01990	0.00665	-0.01236
10 mg/L	0.00161	0.00648	-0.00177

Reference

[1] Kong, Y.; Li, X.; Tao, M.; Cao, X.; Wang, Z.; Xing, B. Cation-π mechanism promotes the adsorption of humic acid on polystyrene nanoplastics to differently affect their aggregation: Evidence from experimental characterization and DFT calculation. Journal of Hazardous Materials 2023, 459, 132071, https://doi.org/10.1016/j.jhazmat.2023.132071.

[2] Hong, A.; Tang, Q.; Khan, A.U.; Miao, M.; Xu, Z.; Dang, F.; Liu, Q.; Wang, Y.; Lin, D.; Filser, J.; et al. Identification and Speciation of Nanoscale Silver in Complex Solid Matrices by Sequential Extraction Coupled with Inductively Coupled Plasma Optical Emission Spectrometry. Anal Chem 2021, 93, 1962-1968, https://doi.org/10.1021/acs.analchem.0c04741.
